# A novel point mutation in RpoB improves osmotolerance and succinic acid production in *Escherichia coli*

**DOI:** 10.1186/s12896-017-0337-6

**Published:** 2017-02-13

**Authors:** Mengyong Xiao, Xinna Zhu, Hongtao Xu, Jinlei Tang, Ru Liu, Changhao Bi, Feiyu Fan, Xueli Zhang

**Affiliations:** 1 Tianjin Institute of Industrial Biotechnology, Chinese Academy of Sciences, Tianjin, China; 2 Key Laboratory of Systems Microbial Biotechnology, Chinese Academy of Sciences, 32 West 7th Ave, Tianjin Airport Economic Park, Tianjin, 300308 China; 3 University of Chinese Academy of Sciences, Beijing, China

**Keywords:** Osmotolerance, RpoB, Succinic acid, Sugar transporter, RNA-seq

## Abstract

**Background:**

*Escherichia coli* suffer from osmotic stress during succinic acid (SA) production, which reduces the performance of this microbial factory.

**Results:**

Here, we report that a point mutation leading to a single amino acid change (D654Y) within the β-subunit of DNA-dependent RNA polymerase (RpoB) significantly improved the osmotolerance of *E. coli*. Importation of the D654Y mutation of RpoB into the parental strain, Suc-T110, increased cell growth and SA production by more than 40% compared to that of the control under high glucose osmolality. The transcriptome profile, determined by RNA-sequencing, showed two distinct stress responses elicited by the mutated RpoB that counterbalanced the osmotic stress. Under non-stressed conditions, genes involved in the synthesis and transport of compatible solutes such as glycine-betaine, glutamate or proline were upregulated even without osmotic stimulation, suggesting a “pre-defense” mechanism maybe formed in the *rpoB* mutant. Under osmotic stressed conditions, genes encoding diverse sugar transporters, which should be down-regulated in the presence of high osmotic pressure, were derepressed in the *rpoB* mutant. Additional genetic experiments showed that enhancing the expression of the *mal* regulon, especially for genes that encode the glycoporin LamB and maltose transporter, contributed to the osmotolerance phenotype.

**Conclusions:**

The D654Y single amino acid substitution in RpoB rendered *E. coli* cells resistant to osmotic stress, probably due to improved cell growth and viability via enhanced sugar uptake under stressed conditions, and activated a potential “pre-defense” mechanism under non-stressed conditions. The findings of this work will be useful for bacterial host improvement to enhance its resistance to osmotic stress and facilitate bio-based organic acids production.

**Electronic supplementary material:**

The online version of this article (doi:10.1186/s12896-017-0337-6) contains supplementary material, which is available to authorized users.

## Background

*Escherichia coli* has been extensively developed for bio-based production of a wide variety of organic acids, including succinic acid (SA) [1, 2]. Although high yields of SA have been successfully achieved using *E. coli* as hosts on both laboratory and commercial scales [2–4], cells suffering from osmotic stress during fermentation remains a major barrier for hyper SA production. One of the main causes of osmotic stress is a high initial sugar concentration in the medium, which is beneficial for simplifying the carbon source feeding process. However, induced osmotic pressure also negatively impacts robustness and propagating fecundity of the bacterial cells. Alkali is usually added during SA fermentation to maintain the medium at a neutral pH [4, 5]. SA accumulates as the dissociated form, disodium succinate, which further aggravate the osmotic stress.

The molecular mechanisms underlying the inhibitory effect due to osmotic stress can be summarized in two aspects. First, since sugar molecules cannot freely travel across semi-permeable cell membranes by diffusion, the high concentrations of such external solvents lead to a strong tendency of cytoplasmic water efflux. This dehydration results in shrinkage of the cell volume and malfunction of cell membranes and embedded proteins, leading to osmotic stress [6]. To counterbalance the deleterious effect of osmotic stress, compatible solutes (also called osmoprotectants), such as potassium ions [7], glycine-betaine [8], trehalose [9], glutamate [10], and proline [11] can spontaneously accumulate in cells via *de novo* synthesis or transport from the medium. Compatible solutes are usually impermeable to the cell membrane, less toxic at high internal concentrations, and not easily catabolized [6, 8], which greatly facilitates water remaining within the cytoplasm. In terms of SA production, it was reported that medium supplemented with glycine-betaine or proline improved cell osmotolerance and succinate production in *E. coli* [12] and *Actinobacillus succinogenes* [13]. However, it is worth noting that the osmoprotective effects of these compatible solutes are conditional. For example, it was reported that internal glycine-betaine lost its protective effect in the presence of NaCl concentrations greater than 1 M [14]. Second, inhibition of nutrition uptake might account for the attenuation of cell growth upon external osmolality. Previous studies using an isotopic labeling experiment demonstrated that in the presence of increased osmolality, the activity of nearly all known sugar transport systems in *E. coli* were inhibited, including the glucose phosphotransferase system (PTS), the binding protein mediated maltose transport system, lactose-proton symport system, and melibiose-sodium co-transport system [15]. Sugar transportation defects leading to energy insufficiency could be partially explained by inhibition of DNA replication [16], protein synthesis and respiration [14] under an osmotic stress. It is noteworthy that such inhibitory effects on growth did not lead to cell death because cell growth and metabolic activity were still maintained at a low level [15]. In addition to attenuation of sugar transport, transcriptional repression of genes encoding sugar transporters might also lead to inhibition of sugar uptake. It was experimentally shown that the transcripts abundance of galactitol and maltose transporter genes were drastically downregulated upon NaCl-induced osmotic stress [17], although transcriptional information for other sugar transporters has not been reported.

Our laboratory previously generated an *E. coli* strain, Suc-T110, for SA production that is highly susceptible to osmotic stress. After maintaining Suc-T110 for more than 1400 generations in a medium containing a high sugar concentration (12% w/v glucose), an osmotolerant strain, HX024, was obtained. Genome re-sequencing of HX024 showed that only seven genes had non-synonymous point mutations, including *rpoB* and *agaR*, which encode transcriptional regulators [4]. In this work, we aimed to discover how these two mutations lead to phenotypic changes in osmotolerance.

## Methods

### Strain, medium and growth conditions

Suc-T110, a derivative strain of the *E. coli* Crooks strain (ATCC#8739), was used as the parental strain in this study. Genetically modified derivatives of Suc-T110 are listed in Table 1. During strain construction, cultures were grown aerobically in Luria broth (per liter: 10 g of Difco tryptone, 5 g of Difco yeast extract, and 10 g of NaCl). For homologous recombination via Red recombinase, which is expressed from a temperature-sensitive plasmid (pKD46) [18], *E. coli* cultures were grown at 30 °C to maintain the plasmid. All other cultures were usually grown at 37 °C. Ampicillin (100 mg L^-1^), kanamycin (50 mg L^-1^), and chloramphenicol (34 mg L^-1^) were added when necessary.

**Table 1:** Strains used in this study

Strains	Genotype	Source
Suc-T110	*E.coli* ATCC#8739, Δ*ldhA*, Δ*pflB*, Δ*ptsI*, Ppck*-*galP*, Ppck*-*pck*	[41]
HX024	Suc-T110, Δ*ackA*-*pta*, Ppck*-*aceBA*, Ppck*-*dcuC*, *ΔmgsA*,adaptively evolved for 1440 generations	[4]
RpoBD645Y	Suc-T110:: *rpoB* (D654Y)	In this study
AgaRR109W	Suc-T110:: *agaR* (R109W)	In this study
OV-*LamB*	Suc-T110, Ppck*- *malK*-*lamB*-*malM*	In this study
RpoBD645Y/∆*malEFG*	RpoBD645Y, ∆*malE*-*malF*-*malG*	In this study
RpoBD645Y/∆*malEFGKM* ∆*lamB*	RpoBD645Y, ∆*malE*-*malF*-*malG* ∆*malK*-*lamB*-*malM*	In this study

Ppck* stand for a mutant of the *E. coli* pck promoter which with a G-to-A mutation at position -64 relative to the ATG start codon

### Genetic methods

Gene knock-outs or overexpression mutants were constructed using a previously described two-step recombination method [18]. Red recombinase was used to facilitate chromosomal gene deletion and modulation [19]. All primers used to construction of mutants are listed in Additional file 1: Table S1. For importation of the mutated *rpoB* into Suc-T110, a *cat*-*sacB* cassette was amplified from plasmid pXZ-CS with the primer set *rpoB*-QC-cat-up/*rpoB*-QC-sacB-down, which was used to replace the native *rpoB* gene in the Suc-T110 chromosome via homologous recombination. Then the mutated *rpoB* gene was amplified from strain HX024 with primer set *rpoB*-up/*rpoB*-down, which was used to replace the *cat*-*sacB* cassette via a second recombination event. Since cells containing the *sacB* gene die when grown on sucrose due to the accumulation of levan, transformants containing the mutated *rpoB* gene were selected for resistance to sucrose [18]. The mutated *agaR* gene was cloned into Suc-T110 by a similar method, and a single deletion of the *malEFG* operon as well as a double deletion of the *malEFG* and *malK*-*lamB*-*malM* operons in Suc-T110 were generated using the the same method. For over-expression of *lamB* in Suc-T110, the promoter for the *malK*-*lamB*-*malM* operon was replaced with a constitutive strong promoter Ppck* using the two-step recombination method as mentioned above.

### Growth under normal or osmotic conditions

Fresh colonies were inoculated into 250-mL flasks containing 30 mL of modified NBS mineral salts medium [20] [per liter: 3.5 g of KH_2_PO_4_, 5.0 g of K_2_HPO_4_, 3.5 g of (NH_4_)_2_HPO_4_, 0.25 g of MgSO_4_ · 7 H_2_O, 15 mg CaCl_2_ · 2H_2_O, 0.5 mg of thiamine, 10.0 g of KHCO_3_ and 0.15 g of betaine HCl and 1 ml of trace metal stock]. The trace metal stock was prepared in 0.1 M HCl and contained the following (per liter):1.6 g of FeCl_3_, 0.2 g of CoCl_2_ · 6H_2_O, 0.1 g of CuCl_2_, 0.2 g of ZnCl_2_ · 4 H_2_O, 0.2 g of NaMoO_4_, 0.05 g of H_3_BO_3_. The medium contained 20 gL^-1^ glucose, and cultures were grown at 37 °C and 250 rpm for 12 h. Cultures were then transferred to 500-mL fermentation vessels, which contained 250 mL of NBS mineral salts medium supplemented with 50 gL^-1^ or 120 gL^-1^ glucose to represent the non-stressed or osmotic stressed conditions, respectively. Then, the cultures were incubated anaerobically for 96 h at 37 °C and 150 rpm. Cell mass was estimated by measuring the optical density at 550 nm (OD_550_) as described previously [9]. The succinate and glucose concentration in the medium were measured by high-performance liquid chromatography according to a previously reported protocol [21].

### RNA extraction

After 48 h of fermentation under normal (5% w/v glucose) or osmotic-stressed (12% w/v glucose) conditions, samples of Suc-T110 and RpoBD645Y were harvested for total RNA isolation. Extraction and additional on-column DNase I treatment was performed with the RNeasy mini kit (Qiagen) according to the manufacturer’s protocol. The purified RNA was assessed with an Agilent 2100 Bioanalyser (Agilent) and quantified using a NanoDrop ND-1000 spectrophotometer (NanoDrop Technologies).

### RNA sequencing and data analysis

Four 90-nt paired-end RNA-seq libraries were generated at Beijing Genomics Institute (BGI, Shenzhen, China) with the HiSeq™ 2000 platform. Quality control of sequencing reads was performed with the NGS QC Toolkit (version 2.3.3) [22], and the obtained high-quality reads were aligned to the *E. coli* ATCC#8739 genome (GenBank CP000946.1) with Bowtie 2 (version 2.2.5) [23]. The aligned reads stored in SAM format file and the raw counts for reads mapping to unique gene were then tallied with HTSeq-count scripts (0.6.0) with the intersection-nonempty resolution mode [24]. Abundance for each transcript was calculated using the Reads Per Kilobase per Million (RPKM) measure as described previously [25]. Differential expression gene calling was performed with R package NOISeq (version 2.6.0) [26] with “probability of differential expression (q value)” ≥0.9 and |log2 Ratio| ≥ 1 as the final cut-off. MIPS FunCat online tools [27] were used to annotate genes with altered expression, and enriched categories (*P* value < 0.001) were marked out.

### Statistical significance tests

Unless otherwise noted, all experiments were performed in triplicate, and statistical tests for significance were determined via a one-way ANOVA using R (version 3.1.1).

## Results and Discussion

### A point mutation in *rpoB* confers resistance to osmotic stress

**Fig. 1:**
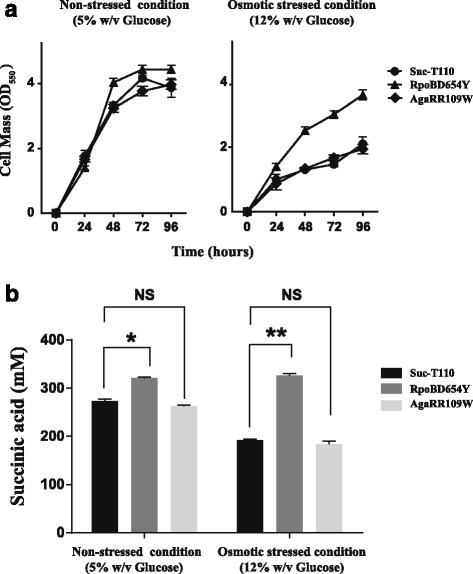
A point mutation in *rpoB* conferred improved cell growth and succinic acid production to Suc-T110 under osmotic stress. **a** Cell growth of RpoBD645Y (A Suc-T110 derivative harboring *rpoB* ^*G1960T*^ [RpoB^D654Y^]), and AgaRR109W (A Suc-T110 derivative harboring *agaR* ^C325T^ [AgaR^R109W^]) along with the parental strain, Suc-T110, under normal (5% w/v glucose) or osmotic stress (12% w/v glucose) conditions. **b** SA production by RpoBD645Y, AgaRR109W, and Suc-T110 after 96 h of fermentation. Data are the mean with the standard error of the mean (SEM, *n* = 3). The significance of differences was calculated by one-way ANOVA; asterisks indicate a significant difference from the control (** *P* < 0.01; * *P* < 0.05; NS = not significant)

As stated above, the osmotolerant mutant HX024 had single, non-synonymous point mutations in both *rpoB* (DNA sequence change, G1960T; Protein sequence change, D654Y) and *agaR* (DNA sequence change, C325T; Protein sequence change, R109W). Given that the physiological functions of the two encoded proteins are both associated with transcriptional regulation, we speculated that the two mutations are likely to cause phenotypic changes in osmotolerance. To test our hypothesis, the coding regions of the mutated *rpoB* and *agaR* were separately amplified from HX024 and were used to replace the corresponding non-mutated genes in Suc-T110. The obtained mutation strains were designated as RpoBD645Y [Suc-T110::*rpoB* (D654Y)] and AgaRR109W [Suc-T110::*agaR* (R109W)]. When cultured in 5% w/v glucose, no obvious growth difference was detected for either RpoBD645Y or AgaRR109W in comparison to Suc-T110 (Fig. 1a). However, under high osmolarity conditions (12% w/v glucose), the growth of both AgaRR109W and Suc-T110 significantly decreased to approximately 45% of that under normal conditions at 96 h, whereas RpoBD645Y showed normal growth under these conditions, i.e., similar to growth in 5% w/v glucose (Fig. 1a). In addition, under high osmolarity conditions, only RpoBD645Y showed a similar SA production titer after 96 h of fermentation as normal growth condition, whereas the other two strains showed lower production (Fig. 1b). These results suggested that the mutated *rpoB* but not the mutated *agaR* rendered Suc-T110 osmotolerant.

In *E. coli* ATCC#8739, the gene *rpoB* (EcolC_4038) encodes the β-subunit of DNA-dependent RNA polymerase (RNAp). RNAp, which is the primary enzyme responsible for gene transcription in prokaryotes, consists of five subunits (α2ββ’ω) and is associated with one of several alternative sigma (σ) factors. Sigma factors, such as σ^S^ in *E. coli* [28], σ^B^ in *Listeria monocytogenes* [29], and RpoN in *Campylobacter jejuni* [30], have been reported to be involved in the regulation of osmotic stress responses. However, direct evidence for the involvement of the RNAp β-subunit in osmotolerance has not yet been reported. An analysis of the protein domain architecture of the RNAp β-subunit using SMART online tools [31] showed that the D654Y mutation was located in a Pfam domain (PF10385) called the external one region of the polymerase. However, the function of this domain has not been characterized. Given that the β-subunit, along with the β’ subunits, constitute the active center of RNAp [32], we speculated that the D654Y mutation might lead to a conformational change in the RNAp active site, and therefore, affect the overall gene transcription pattern. It has been shown that point mutations in the β subunit of *Enterococci* RNAp conferred the capability of adaption to drug stress induced by cephalosporin [33], which probably occurs via a similar mechanism.

### Transcriptomic profiling revealed mutated RpoB caused osmotic response genes upregulated under non-osmotic stressed conditions

**Fig. 2:**
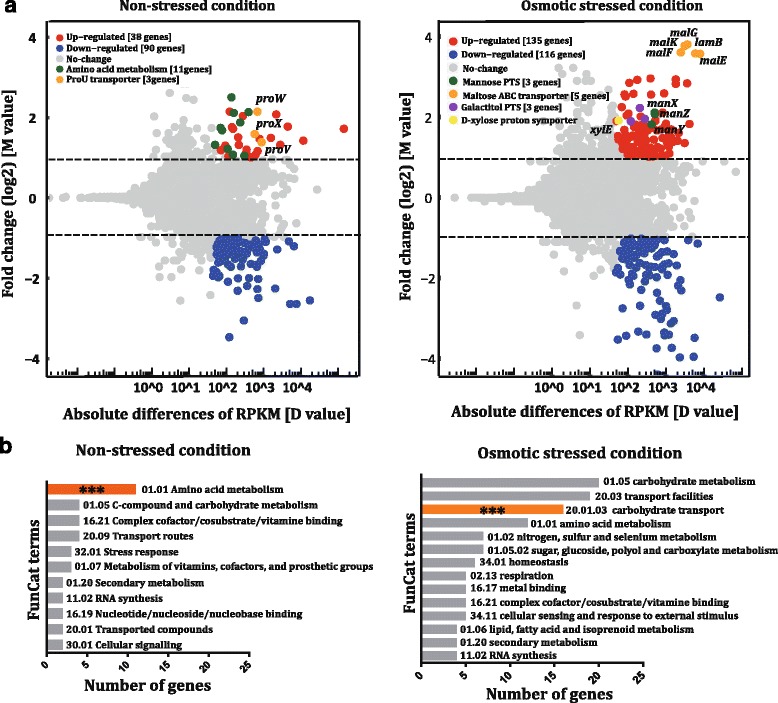
Transcriptional response to osmotic stress in Suc-T110 and RpoBD645Y cells. **a** The altered gene expression patterns elicited by the *rpoB* mutation under either normal (*left panel*) or osmotic stress (*right panel*) conditions were separately plotted in as an MD-plot. M values (Y axis) represent the log2 normalized fold changes. D values (X axis) are the absolute differences in RPKM between Suc-T110 and RpoBD645Y. Each point represents a transcript with a detected expression level. Red or blue points represent differentially expressed genes with increased or decreased abundance, respectively (q value ≥ 0.90 and |log2 ratio| ≥ 1), whereas gray points are genes with no differences in expression. Transcripts used to interpret the osmotolerance phenotype are plotted in different colors. **b** Functional enrichment analysis of the upregulated genes. Typical FunCat terms are listed, and the asterisks (***) indicate a significant enrichment (unadjusted *P*-value < 0.001)

To decipher how the mutated RpoB orchestrated global gene transcription to counteract osmotic stress, RNA-seq analyses were performed on the parental strain Suc-T110 and osmotolerant strain RpoBD645Y under both normal (5% w/v glucose) and high osmolality (12% w/v glucose) conditions (Additional file 2: Table S2, Fig. 2a).

Of the 4200 annotated genes, 128 genes showed altered expression in the strain containing the RpoB D654Y mutation under normal osmolality; with 38 upregulated and 90 downregulated genes. Functional enrichment of the 38 up-regulated genes by MIPS FunCat tools showed that 11 genes were engaged in biological functions of amino acid metabolism (*P*-value = 4.60E-05; Additional file 3: Table S3, P1 and P3, Fig. 2b). As mentioned above, bacterial cells commonly accumulate glutamate and proline to high levels under osmotic stress. The transcriptional abundance of genes involved in the metabolism of glutamate and aspartate (which can be converted into glutamate through transamination [34]), such as EcolC_2869, EcolC_3652, EcolC_3653, EcolC_1833, EcolC_4075, and EcolC_3651, were markedly increased in RpoBD645Y under normal osmolality. Genes involved in proline metabolism were not detected in the up-regulated gene set. However, the expression of genes encoding the major proline transporter system ProU [6, 7] was markedly enhanced. Transcription levels of genes located in the *proU* operon, including *proX* (EcolC_1027, encoding substrate-binding protein), *proW* (EcolC_1028, encoding permease), and *proV* (EcolC_1029, encoding ATP-binding protein), increased 3- to 4- fold in RpoBD645Y. These results were consistent with previous observations that proline accumulation in *E. coli* was due to enhanced transportation and not by synthesis [6, 35]. In addition, the ProU system also functions as the major system for glycine betaine uptake in *E. coli* [36]. A previous study showed that ProU-mediated glycine betaine transport was osmotically stimulated at the level of gene expression [6]. However, to our knowledge, the observation that the *proU* operon highly expressed under non-osmotic stress conditions has not yet been reported. Given that our modified NBS mineral salts medium contains a small amount of betaine (1 mM), we speculated that this compatible solute could be taken up into RpoBD645Y via ProU under non-osmotic stressed conditions, although further study is needed to obtain more direct evidence. In summary, the *rpoB* mutation probably conferred *E. coli* with the ability to mount a “pre-defense” mechanism, such that the osmotic response genes were activated under non-osmotic stressed conditions, which helped the cells better adapt to subsequent osmotic shock.

### Transcriptomic profiling showed that mutated RpoB conferred osmotolerance via derepression of sugar transporters

Under a high osmolality, 244 differentially transcribed genes were identified in RpoBD645Y when compared with Suc-T110; 132 were upregulated and 112 were downregulated (Additional file 3: Table S3, P2). Since genes involved in glutamate synthesis and proline/glycine betaine transportation showed similar transcription levels in RpoBD645Y and Suc-T110 (Additional file 2: Table S2 and Additional file 3: Table S3), strategies other than “pre-defense” mechanisms might be adopted by RpoBD645Y to survive under high osmolality. Functional analysis of 132 upregulated genes indicated that the most significantly enriched physiological function of RpoBD645Y upon osmotic stress was associated with carbon source transportation (FunCat term: 20.01.03 C-compound and carbohydrate transport, *P*-value =9.51E-05; Additional file 3: Table S3, P5, Fig. 2b). This group included genes encoding diverse sugar transporters. The expression levels of these genes have been reported to be drastically reduced under osmotic stress conditions [17, 37, 38]. We found that these sugar transporter genes were drastically repressed in Suc-T110 under osmotic stress, whereas in RpoBD645Y, their expression maintained stable under osmotic stress. For example, transcript levels of *malE* (EcolC_3995), *malF* (EcolC_3996), *malG* (EcolC_3997) *malK* (EcolC_3994), and *lamB* (EcolC_3993), which encode individual subunits of the maltose ABC (ATP-binding cassette) transporter, decreased 10- to 14- fold in Suc-T110 compared to the corresponding levels in RpoBD645Y under osmotic stress. Meanwhile, genes encoding galactose PTS (EcolC_1645, EcolC_1646, and EcolC_1647), mannose PTS (ManY: EcolC_1814 and ManZ: EcolC_1813), D-xylose proton symporter XylE (EcolC_3998), and the melibiose-sodium co-transport system (EcolC_3907) were decreased 2- to 5- fold in Suc-T110 when compared to the corresponding levels in RpoBD645Y. These data demonstrated that repression effects on sugar transport genes were alleviated in RpoBD645Y due to the *rpoB* mutation. Based on FunCat analysis, no other processes with potential osmotic responsive functions were markedly enriched (cut-off, *P* value ≤ 0.001) in RpoBD645Y (Additional file 3: Table S3, P5, Fig. 2b). We assumed that maintenance of sugar transporter gene expression may be helpful for maintaining growth under osmotic stress, which might be the primary reason for the osmotic stress resistance of RpoBD645Y.

### The porin LamB contributes to osmotolerance in RpoBD645Y

**Fig. 3:**
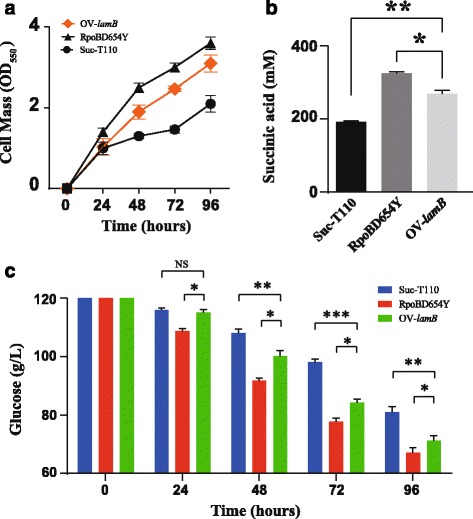
*lamB* overexpression rescued cell growth and the succinate production defect of Suc-T110 under osmotic stress. **a** Growth of *lamB*-overexpressing Suc-T110 (OV-*lamB*) under osmotic stress (12% w/v glucose). **b** SA production of the OV-*lamB* strain after 96 h of fermentation. **c** Glucose consumption by OV-*lamB*. Strains RpoBD645Y and Suc-T110 were used as controls. Data are the mean with the standard error of the mean (SEM, *n* = 3). The significance of differences was calculated with one-way ANOVA; the asterisks indicate a significant difference from the controls (*** *P* < 0.001; ** *P* < 0.01; * *P* < 0.05; NS = not significant)

In *E. coli*, the process of glucose uptake can be divided into two steps. Glucose is first internalized into the periplasm via porins located in the outer membrane, and then imported into the cytoplasm through diverse inner membrane PTS and non-PTS sugar transporters. Three porins including OmpF, OmpC, and LamB, have been reported to be involved in glucose internalization into the periplasm [37]. In addition, the expression of *ompF* and *ompC* in *E. coli* has been shown to be regulated by osmotic stimuli. OmpC predominates at high osmolarity, while OmpF expression is repressed [39], which is consistent with our RNA-seq data (Additional file 2: Table S2). Previous work also demonstrated that OmpC and OmpF were required for cell growth under hyper-osmosis at an alkaline pH and hypo-osmotic stress at an acidic pH; however, they were not required for growth at near neutral pH under both hyper- and hypo-osmosis [39]. These conclusions suggest that OmpC and OmpF might not contribute to osmotolerance of RpoBD645Y because fermentations were performed at a neutral pH. This raised the possibility that osmotolerance may result from the elevated expression of *lamb*. Therefore, we then overexpressed *lamB* in strain Suc-T110. In *E. coli*, *malK* (encoding ATP-binding component of the maltose ABC transporter), *lamB*, and *malM* (encoding periplasmic protein with unclear function) are located in the same operon. For the overexpression experiment, the native promoter of the *malK*-*lamB*-*malM* operon was changed to a strong constitutive promoter Ppck* [20], via homologous recombination to obtain the *lamb*-overexpression mutant OV-*lamB*. Under osmotic stress, we found that the growth and succinate production of this strain were increased 47% and 42%, respectively compared to the corresponding values in Suc-T110 (Fig. 3a and b). In addition, OV-*lamB* had an average glucose consumption rate at 0.5 g L^-1^ h^-1^ during a 96-h fermentation, which was 25% higher than that of Suc-T110 (Fig. 3c). These results suggest that LamB contributed to the osmotolerance of HX024. Previous work demonstrated that LamB contribute about 70% of the total glucose import capacity of the cell under glucose limited conditions [40], suggesting that LamB had a high affinity for glucose under certain conditions. Thus, the contribution of *lamB* overexpression to osmotolerance could be due to enhanced glucose uptake capability under osmotic stress.

### Derepression of the inner membrane-associated maltose ABC transporter confers osmotolerance

In addition to porin proteins, a number of sugar transporters located on the inner membrane were also derepressed in RpoBD645Y under high osmolarity. Thus, additional experiments were carried out to test whether modifying the expression of these transporter genes would alter osmotolerance.

**Fig. 4:**
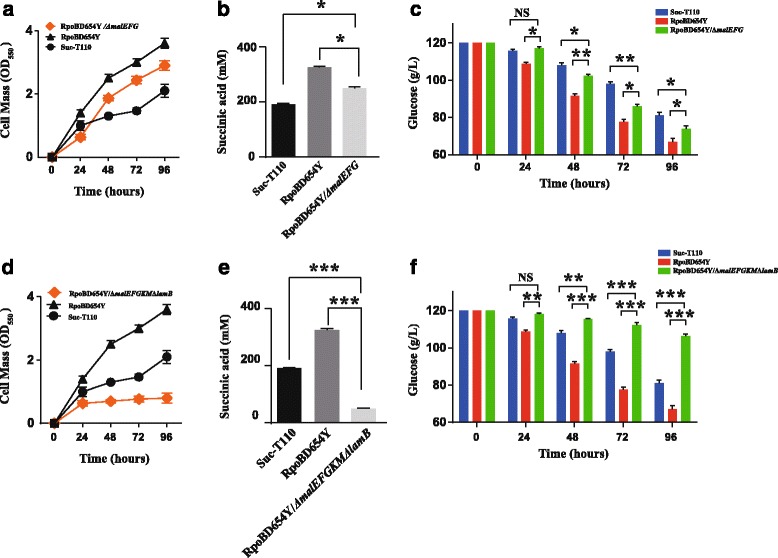
Malfunction of the maltose transporter decreased cell growth and succinate production under hyperosmotic conditions. Growth of (**a**) RpoBD645Y/*ΔmalEFG* and (**d**) RpoBD645Y/∆*malEFGKM*∆*lamB* under osmotic stress (12% w/v glucose). SA production by (**b**) RpoBD645Y/*ΔmalEFG* and (**e**) RpoBD645Y/∆*malEFGKM*∆*lamB* after 96 h of fermentation. Glucose consumption by (**c**) RpoBD645Y/*ΔmalEFG* and (**f**) RpoBD645Y/∆*malEFGKM*∆*lamB* were also measured. Strains RpoBD645Y and Suc-T110 were used as controls. Data are the mean with the standard error of the mean (SEM, *n* = 3). The significance of differences was calculated with one-way ANOVA; the asterisks indicate a significant difference from the controls (*** *P* < 0.001; ** *P* < 0.01; * *P* < 0.05; NS = not significant)

**Fig. 5:**
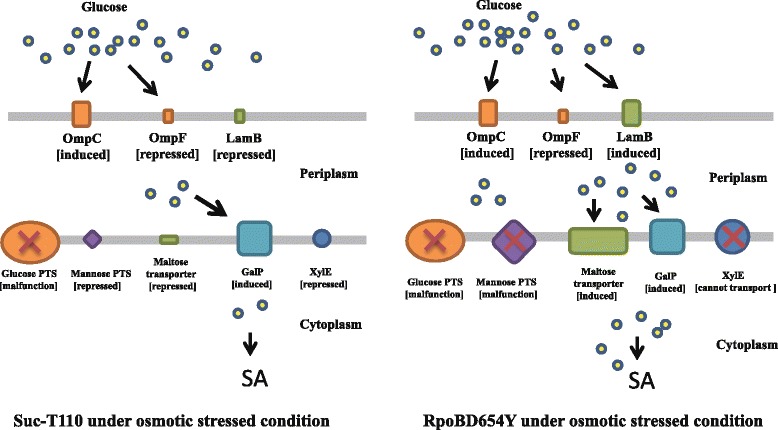
Schematic model illustrating how the D654Y mutation of RpoB affects the osmotolerance of *E. coli* via derepression of sugar transporter genes. Under osmotic stress, the Suc-T110 strain relies on the OmpC porin to transport glucose into the periplasm, which is then transported into the cytoplasm by GalP. However, this transportation process is inefficient. Under osmotic stress in RpoBD645Y, glucose enters cell via both OmpC and derepressed LamB, and then is transported into the cytoplasm by both GalP and derepressed maltose transporter. Transport into RpoBD645Y is more robust due to the presence of more porins and transporters involved in glucose uptake

It should be noted that in Suc-T110, the *galP* gene, which encodes galactose permease, was genetically modified by replacing its native promoter with a strong constitutive promoter Ppck* [41]. In *E. coli*, glucose uptake occurs mainly through the glucose phosphoenolpyruvate: carbohydrate phosphotransferase system (glucose PTS) [42]. However, this internalization process consumes half of the intracellular phosphoenolpyruvate (PEP) for use in glucose phosphorylation, which results in an insufficient PEP supply for SA production [20, 43]. For this reason, the PTS system was impaired in Suc-T110 by deletion of *ptsI* (encoding PTS enzyme I), and GalP was constitutively expressed to restore glucose transport and utilization [38, 44]. Although relatively high *galP* expression was maintained in Suc-T110 even under osmotic stress, cell growth was markedly inhibited (Fig. 1a), suggesting that GalP could not effectively transport glucose under high osmolality. We speculated that this phenotype may be caused by ineffective internalization of glucose into the periplasm. In Suc-T110, LamB and OmpF were repressed, while OmpC was induced under osmotic stress, suggesting that glucose was transported into the periplasm mainly via the OmpC porin. However, OmpC has a smaller pore diameter than other porins [45], suggesting that glucose probably diffused into the periplasm at a lower rate, which would limit the function of GalP. One of the derepressed sugar transporters, D-xylose-proton symporter XylE could not transport glucose [46], and the galactitol and the mannose PTS system became non-functional in Suc-T110 due to the *ptsI* deletion. Thus, only GalP and maltose transporter might contribute to the osmotolerance of RpoBD645Y by enhancing glucose uptake. In *E. coli*, the maltose regulon consists of ten genes that are located on five operons [47]. To obtain genetic evidence that the maltose ABC transporter contributed to the osmotolerance of RpoBD645Y, the *malEFG* operon, which encodes the core subunits of the maltose transporter, was deleted in RpoBD645Y. Cell growth and succinate production of the resulting strain, RpoBD645Y/*ΔmalEFG*, decreased by 24% (Fig. 4a) and 20%, respectively (Fig. 4b), compared with that of RpoBD645Y after 96 h of fermentation. The average glucose consumption rate of RpoBD645Y/*ΔmalEFG* was 0.48 g L^-1^ h^-1^, which was approximately 20% lower than that of Suc-T110 (Fig. 4c). Since LamB usually functions synergistically with the maltose ABC transporter, the adjacent operons of *malEFG and malK*-*lamB*-*malM* were deleted in RpoBD645Y. Cell growth and succinate production of this double deletion mutant (RpoBD645Y/*ΔmalEFGKMΔlamB*) were significantly lower than those of RpoBD645Y, which was even worse than Suc-T110 (Fig. 4d, e). The average glucose consumption rate of RpoBD645Y/*ΔmalEFGKMΔlamB* was only 0.14 g L^-1^ h^-1^, which corresponded to 34% and 25% of the rates of Suc-T110 and RpoBD645Y, respectively (Fig. 4f). This suggested that derepression of the *mal* regulon was involved in the osmotolerance of RpoBD645Y (Fig. 5).

## Conclusion

A novel point mutation (D654Y) within RpoB was identified in this work, which improved the osmotolerance of *E. coli*. This mutation affected the transcriptional activity of RpoB, leading to upregulation of several osmotic response genes which involved in the biosynthesis or transportation of compatible solutes under non-osmotic stressed conditions, probably contributes to compatible solute accumulation. This mutation also enhanced glucose uptake under high sugar osmolality via derepression of the *mal* regulon. Thus, this mutation can be used to improve cell growth under osmotic stress and increase the production of succinate and other organic acids.

## Additional files

Additional file 1: Table S1. Primer list. (DOCX 18 kb)
Additional file 2: Table S2. RNA-seq read profiles mapped to the genome of *E.coli* ATCC#8739. (*XLSX 532 kb*)
Additional file 3: Table S3. Differential expression analysis and functional groups of genes with altered expression. (XLSX 953 kb)

## Abbreviations

ATP: Adenosine triphosphate
EMP: Embden-Meyerhof-Parnas (EMP) glycolytic pathway
G6P: Glucose-6-phosphate
PEP: Phosphoenolpyruvate
PTS: Phosphoenolpyruvate, carbohydrate phosphotransferase system
RNAp: DNA-dependent RNA polymerase
RPKM: Reads Per Kilobase per Million
rTCA: Reductive tricarboxylic acid cycle
SA: Succinic acid
