# Supplementary material for: A novel point mutation in RpoB improves osmotolerance and succinic acid production in Escherichia coli
Source: BMC Biotechnol. 2017 Feb 13;17:10. doi: 10.1186/s12896-017-0337-6 (PMC5307762; doi:10.1186/s12896-017-0337-6)
Supplement: Additional file 1: Table S1. — Primer list. (DOCX 18 kb) [file 12896_2017_337_MOESM1_ESM.docx]

**Supplementary information**

**Table S1 Primers used in this study**

| Primer name | Sequence |
| --- | --- |
| Importation of mutated *agaR* into Suc-T110 | |
| agaR-QC-cat-up | GCTGTGCGTGCCTATGGTGGCGCGTTGATCTGCGATAGCACGACGCCGTCAGTCGAGCCATGTGACGGAAGATCACTTCGCA |
| agaR-QC-sacB-down | CGCAAATGCCCGCCGGTCATCAGCAGCTCAACGCCTTCCGCTTCCAGCAACGCATTAGCTTATTTGTTAACTGTTAATTGTCCT |
| agaR-up | ATGAGTAATACCGACGCTTCAG |
| agaR-down | CCGCAACTTCGCACATCC |
| Importation of mutated *rpoB* into Suc-T110 | |
| rpoB-QC-cat-up | CAGACTAACGAATACGGCTTCCTTGAGACTCCGTATCGTAAAGTGACCGACGGTGTTGTAACTGACTGTGACGGAAGATCACTTCGCA |
| rpoB-QC-sacB-down | CAGTACCAACCAGCGGCTTATCAGCACGCAGAGTCGGAACGGCCTGACGTTGCATGTTCGCACTTATTTGTTAACTGTTAATTGTCCT |
| rpoB-up | CCCTGATGCCTCAGGATATG |
| rpoB-down | AGATGTCGATACCTGCTTCAC |
| Deletion of *malEFG* opreon | |
| malE-QC-cat-up | GAGTCCGTTTAGGTGTTTTCACGAGCACTTCACCAACAAGGA CCATAGATTTGTGACGGAAGATCACTTCGCA |
| malE-QC-sacB-down | GGGATAACGTAAGTTGAGGGTGCAGCGGCATAACATTGGCAGAACAACATCTTTATTTGTTAACTGTTAATTGTCCT |
| malEFG-QC-1 | TCATCGACAGCAACATTCATGATG |
| malEFG-QC-2 | GCTAGGTACCAATCTATGGTCCTTGTTGGTG |
| malEFG-QC-3 | GCTAGGTACCAGATGTTGTTCTGCCAATG |
| malEFG-QC-4 | CTAAGGTAATCGAAAATATATAAC |
| Over-expression of *lamB* | |
| malK-up-cat | AGCCCATCATGAATGTTGCTGTCGATGACAGGTTGTTACAAAGGGAGAAGGGCTGTGACGGAAGATCACTTCGCA |
| malK-down-sacB | CCACGACCTCGCCCCAGGCTTTCGTTACATTTTGCAGCTGTACGCTCGCCATTTATTTGTTAACTGTTAATTGTCCT |
| malK-pck*-F | AGCCCATCATGAATGTTGCTGTCGATGACAGGTTGTTACAAAGGGAGAAGGGCAGCGTGGTGAATCGATACTTTAC |
| malK-pck*-R | CCACGACCTCGCCCCAGGCTTTCGTTACATTTTGCAGCTGTACGCTCGCCATTTCACTGCTCCTTAGCCAATATG |
| Double deletion of *malEFG and malK-lamB-malM* opreons | |
| malM-cat-up | CAACCGTAGGGGCATCACCGTCCAGGATGCCAGGAACGGTGATGTTTTCCTTTACTGTGACGGAAGATCACTTCGCA |
| malG-sacB-down | GGGATAACGTAAGTTGAGGGTGCAGCGGCATAACATTGGCAGAACAACATCTTTATTTGTTAACTGTTAATTGTCCT |
| malM-QC-1 | TGTAACGCCAGCTCCGGCCCGCTTAAC |
| malM-QC-2 | GCTAGGTACCGTAAAGGAAAACATCACCGTTCCTG |
| malEFG-QC-3 | GCTAGGTACCAGATGTTGTTCTGCCAATG |
| malEFG-QC-4 | CTAAGGTAATCGAAAATATATAAC |
